# The expression and function of gelatinolytic activity at the rat neuromuscular junction upon physical exercise

**DOI:** 10.1007/s00418-014-1273-3

**Published:** 2014-09-12

**Authors:** Marine Yeghiazaryan, Anna M. Cabaj, Urszula Sławińska, Grzegorz M. Wilczyński

**Affiliations:** 1 Nencki Institute of Experimental Biology, Polish Academy of Sciences, Pasteura 3, 02-093 Warsaw, Poland; 2 Institute of Biocybernetics and Biomedical Engineering, Trojdena 4, 02-109 Warsaw, Poland

**Keywords:** Matrix metalloproteinase, Exercise, Neuromuscular junction, Nerve, Skeletal muscle, Rat

## Abstract

The gelatinases MMP-9 and MMP-2 have been implicated in skeletal muscle adaptation to training; however, their specific role(s) in the different muscle types are only beginning to be unraveled. Recently, we found that treadmill running increased the activity and/or expression of these enzymes in myonuclei and in activated satellite cells of the soleus (Sol), but not extensor digitorum longus (EDL) muscles on the fifth day of training of adult rats. Here, we asked whether the gelatinases can be involved in physical exercise-induced adaptation of the neuromuscular compartment. To determine the subcellular localization of the gelatinolytic activity, we used high-resolution in situ zymography and immunofluorescence techniques. In both control and trained muscles, strong gelatinolytic activity was associated with myelin sheaths within intramuscular nerve twigs. In EDL, but not Sol, there was an increase in the gelatinolytic activity at the postsynaptic domain of the neuromuscular junction (NMJ). The increased activity was found within punctate structures situated in the vicinity of synaptic cleft of the NMJ, colocalizing with a marker of endoplasmic reticulum. Our results support the hypothesis that the gelatinolytic activity at the NMJ may be involved in NMJ plasticity.

## Introduction

Endurance exercise (repetitive, prolonged exercise of submaximal intensity) has been recognized as an important stimulus improving muscle contractile properties (Gollnick 1986). Recent evidence indicates that the muscle adaptation to training may involve remodeling of extracellular matrix (ECM) by upregulation of major players of the ECM control network, MMP-2 and MMP-9 (also known as gelatinases). There have been studies showing enhanced level of MMP-2 and MMP-9 in rat (Carmeli et al. 2005) and in human (Rullman et al. 2007, 2009) muscles after endurance exercise. In our previous study, we reported that gelatinase activity of soleus (Sol) is enhanced after repetitive training, e.g., under this condition there is upregulation of MMP-2 in the myonuclei and upregulation of MMP-9 in the cytoplasm of activated satellite cells/myoblasts in Sol [but not in extensor digitorum longus (EDL)] (Yeghiazaryan et al. 2012). It is known, however, that muscle adaptation to training is associated with structural and functional changes in the neuromuscular compartment, including the neuromuscular junction (NMJ) (Nishimune et al. 2014). Because the role of gelatinases in activity-dependent synaptic remodeling in the brain is well established, we wondered whether similar functions can be played by these enzymes at the nerve–muscle synapses. Thus, we performed a detailed morphological analysis of the gelatinolytic activity at the NMJ in rat EDL and Sol muscle.

## Methods

### Animals and ethics statement

A total of eight male Wistar rat, aged between 2 and 3 months (mean weigh 200 ± 50 g), were used in this study. The animals had free access to water and chow pellets and were kept in collective cages (four rats per cage). The research was approved by the First Local Ethics Committee in Poland, according to the principles of experimental conditions and laboratory animal care of European Union and the Polish Law on Animal Protection.

### Running session

Before the experiment, rats from the experimental (trained) group (*n* = 4 animals) were acclimated to the motorized treadmill for 3 days, e.g., the animals were placed on the treadmill without running for 10 min each day. After 3 days of familiarization period, the animals started the running session. At the first day of running, the training program was initiated with a warm-up procedure (at the slower speed)—7 cm/s for 10 min at a 0 % grade. After 10 min of low-speed running, the velocity of treadmill was gradually increased to 20 cm/s and rats were running for 35 min, to complete 45 min of running session. From the second day of the experiments, animals were trained for 45 min/daily, 5 days at 20 cm/s with no interruptions. Control-untrained animals (*n* = 4 rats) were kept in their cages without any type of exercise.

### Tissue processing

The procedure was performed as described (Yeghiazaryan et al. 2012). Briefly, the animals were lethally anesthetized with sodium pentobarbital (60 mg/kg body weight) and perfused with 0.5 % paraformaldehyde in 0.1 M phosphate-buffered saline (PBS), pH 7.2. Immediately after perfusion, the Sol and EDL muscles were carefully dissected out, cryoprotected in 30 % sucrose in 0.1 M PBS, frozen, and cut into 14-μm-thick longitudinal sections. The weak fixation (0.5 % PFA) and subsequent cryoprotection were found to be essential for preserving optimal morphology in subsequent in situ zymography. These procedures do not affect the gelatinase activity, as compared to the sections of the snap-frozen tissue (not shown).

### In situ zymography

The cryostat sections were overlaid with a fluorogenic substrate dye-quenched (DQ) gelatin (Invitrogen/Molecular Probes, Eugene, OR, USA) diluted 1:100 in the DQ buffer and incubated for 2 h at 37 °C. Slides were then rinsed 3 × 10 min in PBS containing 0.01 % *Triton* X-100. Cleavage of the substrate by gelatinases resulted in increase of fluorescence intensity by unblocking of quenched fluorescence. After washing, sections were used for immunohistochemical staining.

### In situ zymography with an immobilized DQ gelatin

The procedure of immobilization of DQ gelatin was followed according to (Cavallo-Medved et al. 2009) with some modifications. DQ gelatin was immobilized on glass coverslips, which were coated with Poly-d-Lysine/Laminin. Briefly, 150 μl solution of Poly-d-Lysine/Laminin in 0.1 M borate buffer containing 25 μg/ml DQ gelatin was dispensed onto a coverslips and incubated overnight in the dark at room temperature in 24-well plate. After washing each well with 1 mL sterile PBS, coated coverslips were dried and placed on top of the cryostat sections that had been pre-wetted with a drop of the gelatinase reaction buffer (0.05 M Tris–HCl, 0.15 M NaCl, 5 mM CaCl_2_, pH = 7.6). The specimens were incubated in a pre-heated humid, dark chamber for 2 h at 37 °C. To localize NMJ, Alexa 555 fluorophore-conjugated α-bungarotoxin (αBt) (Invitrogen/Molecular Probes) had been added to the reaction buffer. After the incubation, the specimens were immediately examined under the fluorescent microscope.

### Immunohistochemistry

Immunolabelling was carried out after in situ zymography, as described (Yeghiazaryan et al. 2012). Goat polyclonal anti-calreticulin, and anti-golgin antibodies were a gift from Dr. Marek Michalak from the University of Alberta (Canada). The anti-calreticulin antibody labels endoplasmic reticulum with high specificity, whereas the anti-golgin antibody detects Golgi structure. To visualize nerves and to analyze their internal architecture, we used antibodies against neurofilament (marker of axons) (Dako, Gdynia, Poland), S100β protein (marker of Schwann cells) (Sigma-Aldrich, Poznan, Poland), or myelin basic protein (MBP, marker of myelin sheath) (Sigma-Aldrich). To label presynaptic structure we used anti-synaptophysin antibody (Dako, Gdynia, Poland). To localize NMJ, the specimens were co-stained with an Alexa 488 or 555 fluorophore-conjugated αBt (Invitrogen/Molecular Probes). To visualize basement membrane, a monoclonal mouse anti-β-dystroglycan was used, 1:10 (Novocastra). The immunoreactions were visualized using species-specific secondary antibodies, coupled with a range of Alexa fluorophores (Alexa 488 or Alexa 555 or Alexa 647, all from Invitrogen/Molecular Probes), diluted 1:200. Nuclei were counterstained with 4,6-diamidino-2-phenylindole (DAPI) or TO-PRO-3 (Invitrogen/Molecular Probes).

### Microscopy and image processing

The fluorophore-labeled specimens were examined under the Leica TCS SP5 confocal system, or Leica DRB wide field fluorescence microscope equipped with a digital camera. The quantitative analysis was performed using Fiji (http://fiji.sc/). To measure the gelatinolytic activity at the NMJ, every image-stack, in which red channel represented αBt, green channel represented DQ gelatin signal, blue channel represented the staining with a mixture of anti-neurofilament and anti-synaptophysin antibodies, and white channel represented DAPI staining, was split into four individual stacks, corresponding to different channels. The red, blue, and white stacks were thresholded, binarized, and inverted, to create 3D masks corresponding to each of the three aforementioned structural components of the NMJ. The mean intensity of green channel was then measured within the volume occupied by each mask. The mean junctional activity ± standard error was calculated for every experimental group (for each animal up to 10 NMJ). The results were statistically analyzed using Student *t* test.

Highest resolution confocal images were restored by three-dimensional (3D) deconvolution using Huygens Professional software, (Scientific Volume Imaging, http://www.svi.nl/) by applying classic maximum-likelihood estimation algorithm and automatically generated point-spread function. For final inspection, the images were processed using Corel Package (Corel Corporation, Ottawa, Ontario, Canada). The three-dimensional reconstructions were done using Imaris (Bitplane, http://www.bitplane.com/).

The degree of colocalization between DQ gelatin and calreticulin or golgin was assessed by calculating the Pearson’s correlation coefficient in the Fiji image analysis software using the JACoP plugin. The coefficient can vary between −1 and +1, values close +1 indicate colocalization, while values close to 0 indicate lack of correlation between the two signals.

The area of colocalization between immobilized DQ gelatin and αΒt was highlighted with Fiji function Colocalization Threshold.

### Statistical analysis

Student *t* test was used for statistical evaluation of differences between control and trained, treated and untreated groups. Results were expressed as mean ± standard error of the mean (SEM). In all analyses, statistical significance was set at *p* < 0.05.

## Results

### The gelatinolytic activity at the NMJs of EDL increases upon training

To address the question of whether exercise is associated with changes in the gelatinolytic activity of the nerves and neuromuscular synapses, we performed colocalization experiments, in which in situ zymography was followed by specific markers of nerves and NMJs, in both EDL and Sol muscles of control- and 5 days-trained rats.

**Fig. 1:**
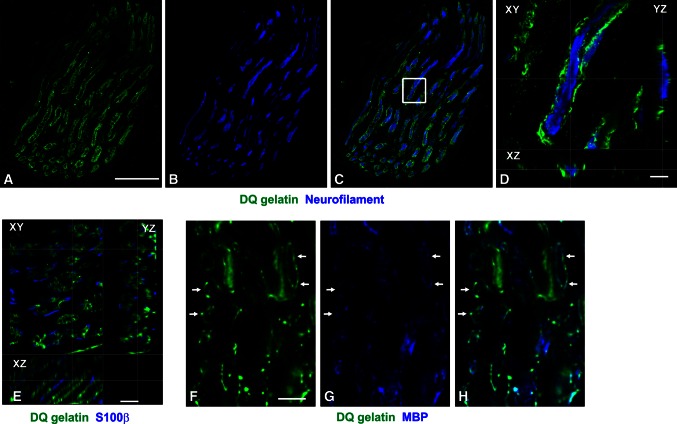
In situ zymographic analysis of gelatinase activity in the intramuscular nerve twigs. Confocal micrographs of EDL sections after 5 days of locomotor training, double fluorescent stained for gelatinase activity (*green* **a**, **c**, **d**, **e**, **f**, **h**), and neurofilament (*blue* **b**–**d**) or S100β (*blue* **e**), or MBP (*blue* **g**, **h**) immunoreactivities. **a**–**c** Maximum projection images. *Scale bars* 30 μm. **d** An enlarged view of the area highlighted in **c** by a *white rectangle*; shown are single-plane cross sections through the confocal stack. Note that the axon is surrounded by gelatinolytic activity, but itself is devoid of the activity. *Scale bar* 5 μm. **e** Single-plane cross sections through the confocal stack demonstrating that S100β-positive Schwann cells cytoplasm does not contain gelatinolytic activity. *Scale bar* 5 μm. **f**–**h** Single-plane confocal images demonstrating the colocalization of gelatinolytic activity (*green*) with MBP-immunoreactive myelin (*blue*). *Scale bar* 4 μm

In both, Sol and EDL muscles, the intramuscular nerve twigs were associated with strong gelatinolytic activity (Fig. 1), which, however, was not changed by training. Within the nerves, the activity was found to surround individual axons, whereas both, axons themselves and Schwann cell cytoplasm, were devoid of the activity (Fig. 1a–e). By colocalization with the MBP immunoreactivity, the gelatinase-positive layer around axons was identified as myelin sheath (Fig. 1f–h). It should be noted, however, that the colocalization of the gelatinolytic activity and MBP was evident only in small nerve twigs, having thin myelin sheats. Within large nerves, the layer of the gelatinolytic activity around axons was MBP-negative, most probably as a results of the incomplete penetration of the anti-MBP antibody (not shown).

**Fig. 2:**
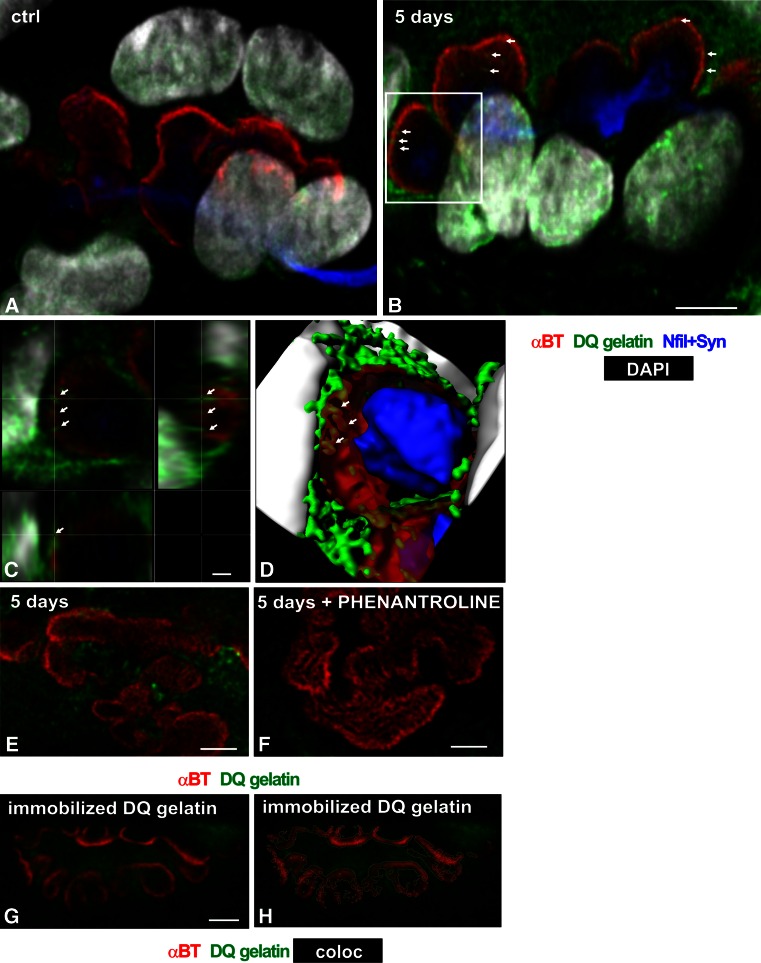
The pattern of gelatinolytic activity at the NMJ of control and trained rat EDL muscle. **a**–**c** Triple fluorescent staining for gelatinase activity (*green*), αBt (*red*), synaptophysin and neurofilament (*blue*) demonstrating that NMJs of control rat contain very little gelatinolytic activity (**a**); training results in an increase of enzymatic activity at the postsynaptic domain of the NMJ (**b**); at some sites, the gelatinase-positive punctate objects are present within αBt-positive postsynaptic folds (*arrows*). Nuclei identified by DNA-specific staining (DAPI, *white*). The images are confocal maximum projection of a few Z planes. *Scale bar* 10 μm. **c** High-magnification image of substack outlined by a *white rectangle* in (**b**). Note that enhanced gelatinolytic activity forms finger-like protrusions directed toward, and colocalizing with, the area of postsynaptic folds as shown by single-plane cross sections of the entire confocal stack in all three dimensions (*XY*, *XZ and*, *YZ*). *Scale bar* 1 μm. **d** 3D reconstruction image of the substack shown in (**c**) demonstrating that the gelatinolytic activity (*green*) fills the junctional folds (*reddish*, transparent). **e**, **f** Control experiment: the sections were incubated in the reaction buffer without (**e**) or with (**f**) phenantroline. **g**, **h** Control experiment, in which DQ gelatin was immobilized on glass surface, to rule out its differential binding to various tissue constituents; the DQ signal (*green*) remains associated with αBt-stained NMJ (*red*), but its intensity decreased; In **h**, the area of colocalization between the *red* and *green* channels is outlined in *white*, using the Fiji (http://fiji.sc/) function Colocalization Threshold. *Scale bars* 1 μm

In control muscles, NMJs were found to contain very little gelatinolytic activity which, besides nuclei, was present as a reticular-like structure at the postsynaptic domain, extending into the surrounding sarcoplasm. Notably, in the exercised EDL, but not Sol, the postsynaptic reticular activity appeared to be enhanced (Fig. 2a–d). A detailed morphological analysis of this enhanced activity at high magnification revealed that it formed finger-like protrusions directed toward the area of postsynaptic folds labeled with αBt (Fig. 2c, d). Usually the tips of these protrusions colocalized with αBt signal, thus, in single optical sections, they often appeared as tiny dots. The activity was found neither at the presynaptic compartment (labeled with a mixture of anti-synaptophysin and anti-neurofilament antibodies) nor within the terminal Schwann cells (labeled for S100β).

The specificity of in situ zymography was controlled by the use of a zinc chelator 1, 10-phenanthroline, which is the general metalloproteinase inhibitor. To exclude the possibility that the observed localization of the gelatinolytic activity results from differential absorbance of DQ gelatin to various structures in the section, we performed an experiment in which the coverslip had been pre-coated with DQ gelatin according to (Cavallo-Medved et al. 2009). The signal occurred to be weaker, but its distribution was the same as with liquid DQ gelatin (Fig. 2g, h).

**Fig. 3:**
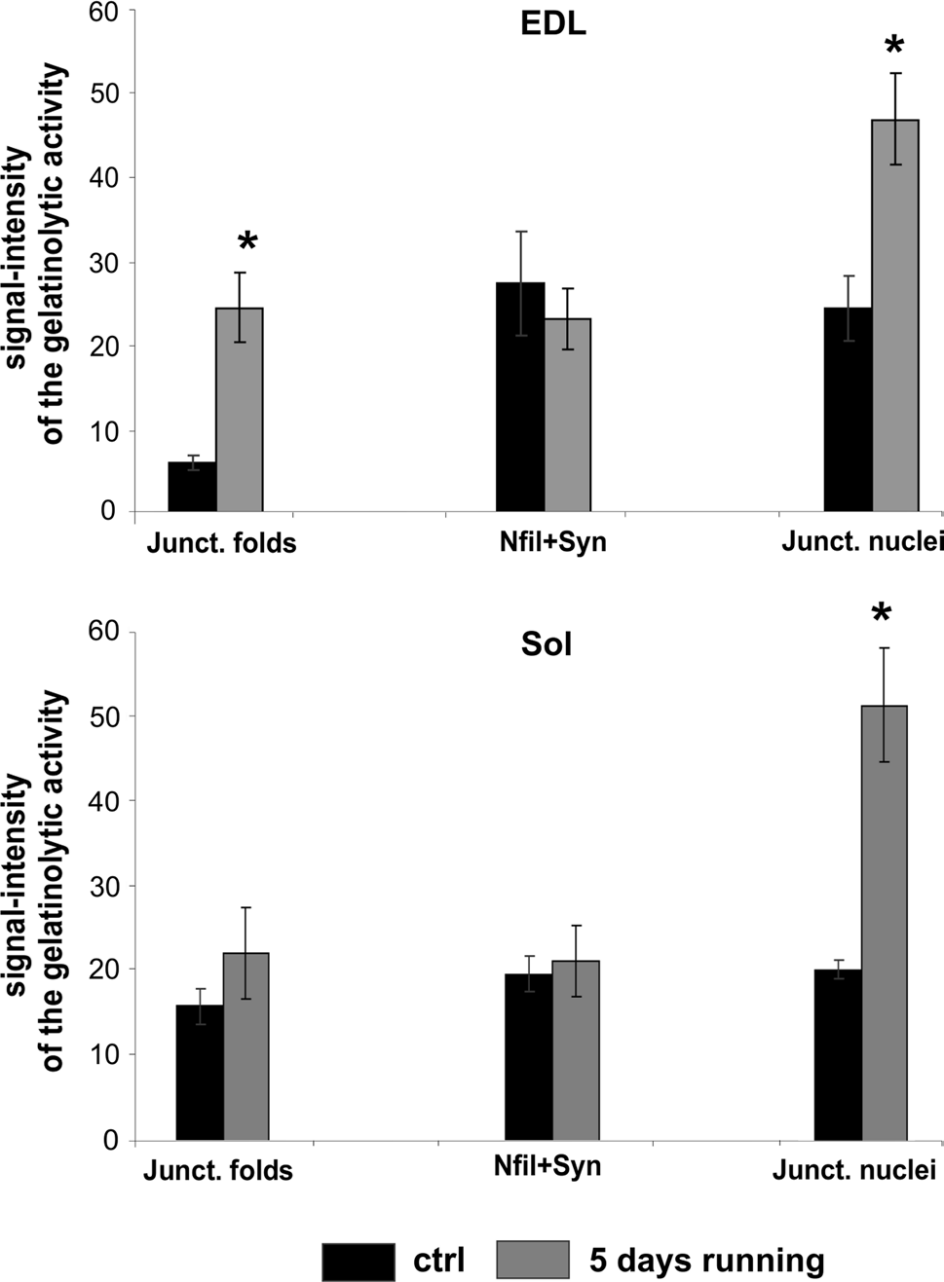
Quantitative evaluation of gelatinase activity at the NMJ in the muscle fibers of EDL (*upper panel*) and Sol (*lower panel*) after 5 days of treadmill running. The signal intensities were measured in junctional folds, synaptic boutons and terminal axon branches (identified by neurofilament and synaptophysin), and in junctional nuclei in control (*black bars*) and trained (*gray bars*) rats. Data for each column were obtained from four male rats (the numbers of NMJ observed during experiments were: *n* = 31 for EDL, *n* = 29 for Sol). Signal intensity is equal to the *gray* level value in 8-bit channel image. *Error bars* represent standard error. *Asterisk* indicates statistical significance, compared to control, *p* < 0.05 by Student *t* test

The quantitative intensity analysis of the gelatinolytic activity at the postsynaptic folds of EDL and Sol muscle in control and trained animals confirmed that the postsynaptic area of the former contained more activity in the rats trained for 5 days than in control rats (Fig. 3). Thus, it seems that at the circumscribed area of NMJs, the exercise-induced changes were opposite to the bulk of the muscle (Yeghiazaryan et al. 2012).

**Fig. 4:**
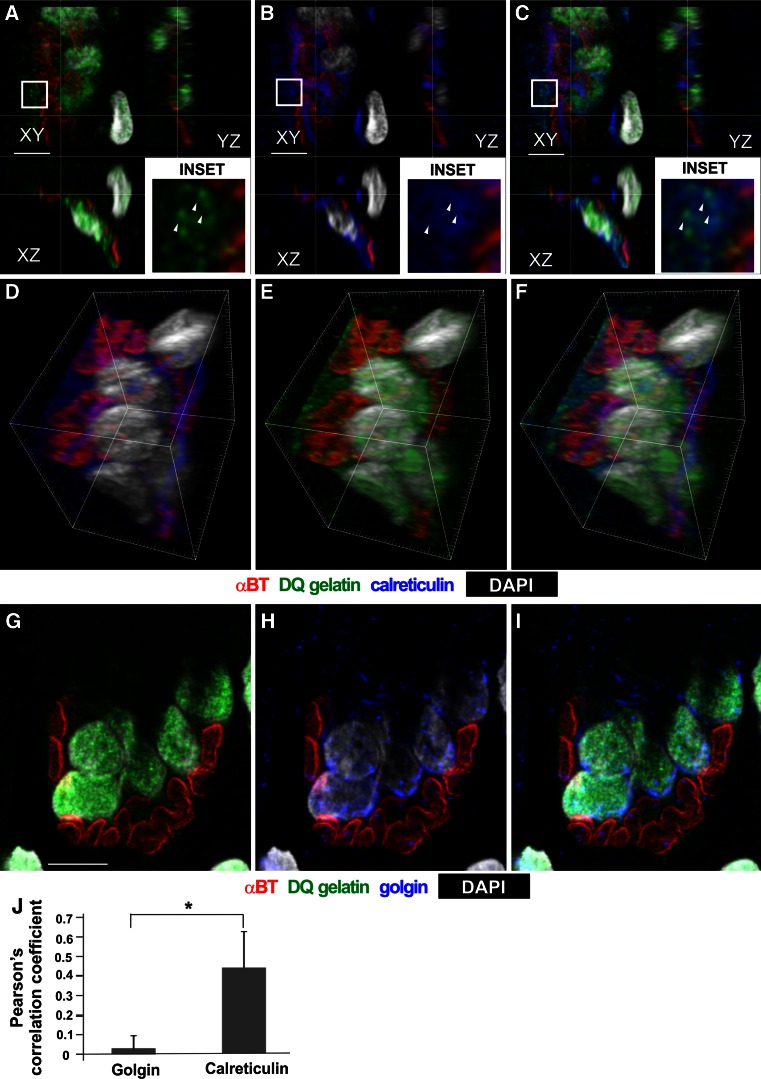
Analysis of gelatinase activity in the endoplasmic reticulum and Golgi apparatus of trained EDL muscle. **a**–**c** High-magnification confocal images of EDL NMJ labeled for gelatinase activity **(** *green* **a**, **c**), αBt (*red* **a**–**c**) and calreticulin (*blue* **b**, **c**). Nuclei labeled with DAPI (*white*). Note the partial colocalization between *green* and *blue* signal in **c**. *Arrowheads* in *insets* point to the gelatinase-positive puncta that are calreticulin-positive. **d**–**f** 3D reconstructions of confocal stack showing a wide area of overlap between gelatinolytic activity and endoplasmic reticulum marker. **g**–**i** High-magnification confocal images of EDL NMJ labeled for gelatinase activity (*green* **g**, **i**), αBt (*red* **g**–**i**) and golgin (*blue* **h**, **i**). **j** Quantitative analysis demonstrating that the colocalization of gelatinolytic activity with golgin is negligible compared to the colocalization with calreticulin. *Scale bars* 10 μm

To reveal the subcellular identity of the gelatinase-positive reticular structure and its finger-like protrusions, we investigated whether it colocalizes with the markers of endoplasmic reticulum (calreticulin) or Golgi apparatus (golgin). The quantitative analysis demonstrated partial colocalization of the gelatinolytic activity with calreticulin (Fig. 4) (Pearson coefficient 0.43) and virtually no colocalization with golgin (Pearson coefficient 0.03) (Fig. 4).

However, the punctate gelatinase-positive objects, representing the tips of finger-like protrusions embedded within the postsynaptic folds, contained neither marker (Fig. 4, arrowheads).

**Fig. 5:**
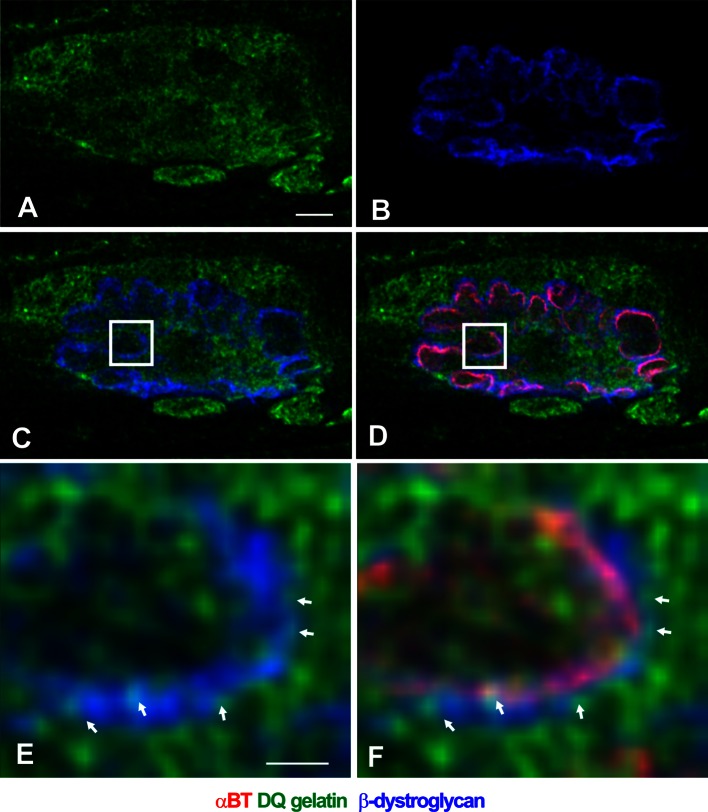
Colocalization of gelatinolytic activity with β-dystroglycan. **a**–**d** Low-magnification confocal images of EDL NMJ labeled with DQ gelatin (*green*) and immunostained for β-dystroglycan (*blue*). In (**d**) the postsynaptic folds were visualized with a-BT (*red*). *Insets* in **c**, **d** show the position of the area that was magnified in (**e**, **f**). *Arrows* in (**e**, **f**) point to the sites of colocalization situated around postsynaptic folds. *Scale bars* (**a**–**d**) 10 μm, (**e**, **f**) 1 μm

To determine whether β-dystroglycan can be a substrate for gelatinases after physical training, we co-immunostained the in situ zymography EDL specimens from 5-day-trained animals with the anti-β-dystroglycan antibody. We found distinctive colocalization pattern between the two signals around the receptor zone at the NMJ (Fig. 5). The colocalization is restricted to the β-dystroglycan-containing plasma membrane infoldings and does not occur in case of the intracellular majority of the gelatinolytic activity.

## Discussion

In addition to improvements of oxygen uptake, the endurance exercise result in physiological and morphological changes of NMJ, which have a high degree of structural plasticity. For example, in the study of Andonian and Fahim (1987), it was shown that 30 days of endurance treadmill exercise (for 1 h/day at velocities up to 30 m/min) resulted in an increased nerve terminal area and a number of branches in the EDL and Sol muscles of young adult rats, with greater magnitude of changes in the EDL. Similar results were established by Waerhaug et al. (1992), who described the significant increase in the area and length of the nerve terminals after 6 weeks of treadmill running in EDL, but not in Sol of young adult rats. Other group (Tomas et al. 1997) demonstrated that physiological walk training program lasting 4 weeks (55 min daily at a speed 27 m/min) caused ultrastructural changes in the morphology of the pre- and postsynaptic part of NMJs of the EDL muscle of adult rats which were directly related to neurotransmission in muscles after training. Since it is known, that the structural integrity of NMJ is provided by the specialized zone of cell-associated ECM, or basal lamina, occupying the synaptic cleft (Sanes 2003), the ECM proteolytic remodeling is expected to occur, to allow the changes in junctional structure.

The presence of MMPs at the NMJ is well known. Kherif et al. (1998) were the first to demonstrate the expression of MMP-2 and MMP-9 at mouse NMJ by immunofluorescence (Kherif et al. 1998). Later, also MMP-3 and MMP-7 were localized at the synapses (Schoser and Blottner 1999; VanSaun and Werle 2000). Among MMPs discovered at the NMJ, the role of MMP-3 in controlling synaptic structure and function via regulation of agrin level was established (VanSaun et al. 2003, 2007). Although in previous studies the role of MMP-2 and MMP-9 in the denervation/reinnervation processes was assessed (Demestre et al. 2005; Kherif et al. 1998, 1999), to our knowledge, we were the first to study the changes of gelatinolytic activity at the NMJs in response to training. Notably, the relatively early rise of the activity in our study (after 5 days) suggests that the phenomenon may precede, and mechanistically contribute to, NMJ remodeling that occurs during subsequent weeks of training. Because the activity could be blocked by a zinc chelator 1,10-phenanthroline, the enzymes responsible for gelatinolysis in our study are MMPs, most likely gelatinases.

In our study, the proteolytic activity was upregulated and localized in the postsynaptic part of NMJs of trained muscle. The quantification of signal intensities of gelatinolytic activity at different compartments revealed significant upregulation mainly in the junctional nuclei and synaptic cleft of NMJ of EDL muscle in response to repetitive training. The absence of general upregulation throughout the whole EDL muscle (Yeghiazaryan et al. 2012), but the presence of it at the NMJ can be explained by the fact that the NMJ occupies less that 0.1 % of the surface of individual muscle fibers (Merlie and Sanes 1985). This explains why we were able to detect it only after the detailed analysis. The reason how the significant upregulation of the gelatinolytic activity occurs selectively in EDL, but not in Sol is unclear; however, it corresponds very well to the reported selective enlargement of the endplate in the former, but not in the latter muscle, upon endurance training (Waerhaug et al. 1992).

Although the function of gelatinases at the NMJ is presently unknown, several predictions can be made, based on the literature. The most obvious substrate of gelatinases at the NMJ would be collagen IV and laminins, the key components of the synaptic basal lamina (Singhal and Martin 2011). Collagen IV and laminins can be cleaved by both MMP-2 and MMP-9 (Sternlicht and Werb 2001). One can speculate that the widespread action of gelatinases on the synaptic ECM leads to the weakening of the physical constraints imposed on both pre- and postsynaptic domains, thus enabling their training-induced expansion. Another candidate substrate of MMPs present at the NMJ is agrin. It was demonstrated that agrin can be cleaved by MMP-3 in a synaptic activity-dependent manner (Werle and VanSaun 2003), and that this action is important for maintenance of an appropriate synaptic structure and function (Chao et al. 2013; VanSaun et al. 2003). Recently it was found that MMP-9, but not MMP-2, can also cleave agrin in vitro, at the site that is distinct from the one utilized by MMP-3 (Patel et al. 2012). A candidate substrate of both gelatinases at the NMJ is also β-dystroglycan (Sbardella et al. 2012) (Fig. 5). Interestingly, synaptic activity-dependent cleavage of β-dystroglycan by MMP-9 was demonstrated in neurons (Michaluk et al. 2007). MMP-9 is localized to the postsynaptic domain of brain excitatory synapses, being involved in synaptic plasticity. A study of Michaluk et al. (2009) identified MMP-9, as a physiological regulator of *N*-methyl-d-aspartate (NMDA) receptor surface trafficking. It is known that NMDA receptors are present at postsynaptic membrane of NMJ together with acetylcholine (ACh) receptors (Berger et al. 1995; Mays et al. 2009). Although glutamate is not the primary neurotransmitter at the vertebrate NMJ, it was established that endogenous glutamate is released with quantal ACh from motor nerve terminals (Landry et al. 2004) and may modulate neuromuscular transmission and could be involved in the safety mechanisms at the synapse (Mays et al. 2009).

Here, we observed a strong gelatinolytic activity associated with the intramuscular nerve twigs in both, Sol and EDL muscles, which, however, was not changed by training. Consistent with our results, the presence of MMP-2 and MMP-9 immunoreactivities was reported in the intramuscular nerves of mouse (Kherif et al. 1998), rat (Hughes et al. 1998), and human (Renaud and Leppert 2007) normal skeletal muscles. It was also demonstrated that MMP-9 expression in regenerating nerves was significantly enhanced, after crush (mouse sciatic nerve) and axotomy (rat sciatic nerve) (Hughes et al. 2002; Platt et al. 2003; Shubayev and Myers 2004).

The present study demonstrated the partial presence of gelatinolytic activity in the endoplasmic reticulum of EDL muscle after 5 days of treadmill running. It is unlikely that the intracellular gelatinolytic activity resulted from relocation of gelatinases, e.g., from the basement membrane to the intracellular membranes as an artifact of the cryosectioning. In our previous study, in which the conditions of tissue processing were the same as in the present one, we observed gelatinase-positive myonuclei and gelatinase-negative nuclei of quiescent satellite cells separated from each other by the distances of a few micrometers (Fig. 1 H in Yeghiazaryan et al. 2012). This finding indicates that our conditions of tissue fixation and cryoprotection prevented an artifactual redistribution of the enzymes even at the very small distances. Therefore, our present findings seem to support the evidence of MMPs intracellular activation. Intracellular MMPs activation may be performed by the endoprotease of the convertase family—furin, serin proteases, caspases, and by autolytic cleavages (Cauwe and Opdenakker 2010), and the intracellularly active MMPs may function inside the cells. In this regard, our previous experimental work showed upregulation of active MMP-2, which was colocalized with an activated RNA polymerase II in the myonuclei of trained Sol muscle (Yeghiazaryan et al. 2012). The partial colocalization between expressed gelatinolytic activity and the marker for endoplasmic reticulum and the absence of activity in the Golgi apparatus suggests that at the NMJ the gelatinases may be secreted to the extracellular space via non-classical secretion way (independently of the endoplasmic reticulum-Golgi pathway). The calreticulin-negative gelatinolytic spots located at the acetylcholine receptor zone might represent some kind of exocytotic organelles/vesicles that no longer contain endoplasmic reticulum-resident proteins. Alternatively, this could represent a juxtamembrane gelatinase(s) that is secreted to the synaptic cleft and anchored therein by yet unidentified mechanisms. This fits well to the concept of synaptic podosomes presented by Proszynski et al. (2009), Proszynski and Sanes (2013).

In conclusion, our results suggest that synaptic activity results in the activation of gelatinases at the NMJ and that they may be involved in activity-dependent synaptic plasticity.

## Abbreviations

αBt: α-Bungarotoxin
ACh: Acetylcholine
DAPI: 4,6-Diamidino-2-phenylindole
DQ: Dye-quenched
ECM: Extracellular matrix
EDL: Extensor digitorum longus
MBP: Myelin basic protein
MMP: Matrix metalloproteinase
NMDA: *N*-methyl-d-aspartate
NMJ: Neuromuscular junction
PBS: Phosphate-buffered saline
PFA: Paraformaldehyde
Sol: Soleus
